# The Role of the Surface Acid–Base Nature of Nanocrystalline Hydroxyapatite Catalysts in the 1,6-Hexanediol Conversion

**DOI:** 10.3390/nano11030659

**Published:** 2021-03-08

**Authors:** Asato Nakagiri, Kazuya Imamura, Kazumichi Yanagisawa, Ayumu Onda

**Affiliations:** Research Laboratory of Hydrothermal Chemistry, Faculty of Science, Kochi University, 2-17-47 Asakurahonmachi, Kochi 780-8073, Japan; nkgrpon3@gmail.com (A.N.); imamura-kazuya@kochi-u.ac.jp (K.I.); yanagi@kochi-u.ac.jp (K.Y.)

**Keywords:** hydroxyapatite, acid–base catalyst, hydrothermal synthesis, 1,6-hexanediol, nanocrystalline materials, sodium containing hydroxyapatite

## Abstract

Hydroxyapatite is known to have excellent catalytic properties for ethanol conversion and lactic acid conversion, and their properties are influenced by the elemental composition, such as Ca/P ratio and sodium content. However, few reports have been examined for the surface acid–base nature of hydroxyapatites containing sodium ions. We prepared nanocrystalline hydroxyapatite (Ca-HAP) catalysts with various Ca/P ratios and sodium contents by the hydrothermal method. The adsorption and desorption experiments using NH_3_ and CO_2_ molecules and the catalytic reactions for 2-propenol conversion revealed that the surface acid–base natures changed continuously with the bulk Ca/P ratios. Furthermore, the new catalytic properties of hydroxyapatite were exhibited for 1,6-hexanediol conversion. The non-stoichiometric Ca-HAP(1.54) catalyst with sodium ions of 2.3 wt% and a Ca/P molar ratio of 1.54 gave a high 5-hexen-1-ol yield of 68%. In contrast, the Ca-HAP(1.72) catalyst, with a Ca/P molar ratio of 1.72, gave a high cyclopentanemethanol yield of 42%. Both yields were the highest ever reported in the relevant literature. It was shown that hydroxyapatite also has excellent catalytic properties for alkanediol conversion because the surface acid–base properties can be continuously controlled by the elemental compositions, such as bulk Ca/P ratios and sodium contents.

## 1. Introduction

Hydroxyapatite (Ca-HAP) is a major component of bone and tooth enamel. Ca-HAP has a high thermal stability and biocompatibility. The stoichiometric form of Ca-HAP is shown as Ca_10_(PO_4_)_6_(OH)_2_, and its Ca/P molar ratio is 1.67. However, while maintaining the single phase of apatite crystal structure, it is possible to change the Ca/P ratio ranging from 1.5 to 1.7 and substitute the constituents of Ca^2+^, PO_4_^3−^, and OH^−^ with other elements to some extents [1]. Ca-HAP exhibits various unique properties, depending on changes in the chemical compositions. In recent years, much research has been conducted on the synthesis and application of Ca-HAP, and its application is expected in the fields of adsorbents, catalysts and catalyst supports, composite materials with polymers, biomedical materials, and nanopapers.

Many research examples have been reported in recent years regarding the catalytic properties of Ca-HAP [2,3,4,5,6,7,8,9]. Ca-HAP exhibits highly stable against reaction conditions and no highly acidic and/or base properties, so it is appropriate as a catalyst. These properties are particularly effective in converting oxygen-containing organic compounds, such as alcohols and carboxylic acids, at reaction temperatures of around 300–400 °C. Previous studies revealed a high selectivity of 1-butanol production in ethanol conversion [8,9,10] and a high selectivity of acrylic acid production in lactic acid conversion [11,12,13,14]. Many studies have been conducted on the effects of element types and Ca/P ratios on these reactions [8,9,10,11,12,13,15]. In addition, in lactic acid conversion, the Na-containing HAP catalyst showed a particularly high acrylic acid yield [12]. It has been reported to have catalytic deactivation resistance by the other group [16].

However, although hydroxyapatite is expected to be effective for selective conversion of many oxygen-containing organic compounds, such as alkanediols, there are few reports of attractive catalytic properties. Alkanediol is now produced from petroleum and widely used as a polymer raw material. In recent years, it has been found that alkanediols, such as ethylene glycol, propylene glycol, and hexanediol, were obtained from biomass-derived compounds, such as glucose, [17,18,19,20,21,22] and it is expected as one of basic renewable raw materials for various bulk and fine chemicals in the future to realizing a sustainable society [23,24,25,26].

For use hydroxyapatite as a catalyst with high catalytic activity and high product selectivity, high specific surface area and high surface uniformity are important. In addition, it is expected to change the surface Ca/P ratios of hydroxyapatite nanocrystalline particles. In general, the hydrothermal method is suitable for synthesizing high crystalline nanoparticles. However, in the hydrothermal synthesizing methods, nanoparticles grow from the crystal nucleus vis the repeating of dissolution and precipitation. Therefore, does the surface structure and catalytic properties continuously change in response to changes in bulk Ca/P ratios, or does those surface properties not continuously change but maintain a relatively stable surface? The question about whether to take the structure is not revealed. In particular, there are few studies on the effect of the Ca/P ratio on Na-containing Ca-HAP particles which have unique catalytic properties.

**Scheme 1:**
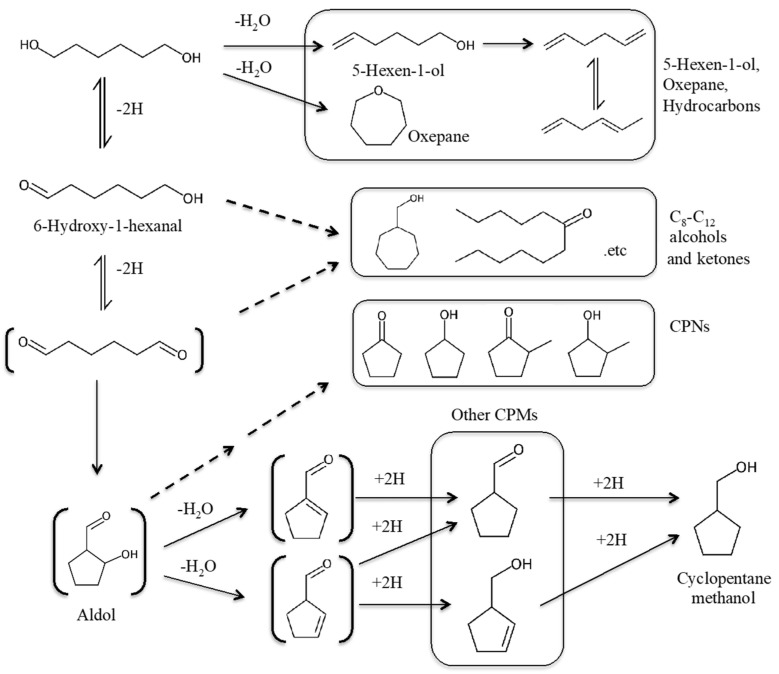
Probable reaction route of 1,6-hexanediol conversion based on reference [24].

In this study, nanocrystalline Na-containing hydroxyapatite catalysts with various Ca/P ratios were prepared by the hydrothermal method, and the effects of Ca/P ratios of the nanocrystalline HAPs on the catalytic properties were examined. The relationship between the acid–base adsorption properties and the catalytic properties of the HAPs with various bulk Ca/P ratio were investigated to clarify whether the surface structures and catalytic properties continuously changes with changes in the bulk Ca/P ratio. In addition, this study first reports the unique catalytic properties of nanocrystalline Ca-HAP in 1,6 hexanediol conversions (Scheme 1). By controlling Ca/P ratios of nanocrystalline Ca-HAP catalysts, the 1,6 hexanediol conversion provides both useful compounds, unsaturated alcohols and cycloalkane alcohols, with higher yields and selectivity than the previously reported catalysts.

## 2. Materials and Methods

### 2.1. Materials

For use in this study, Ca(NO_3_)_2_·4H_2_O, P_2_O_5_, NaOH, SiO_2_, Ca(OH)_2_ and 2-propanol were purchased from Fujifilm Wako Pure Chemical Co., Osaka, Japan. The necessary 1,6-hexanediol and ethanol were purchased from Sigma-Aldrich Co., St. Louis, MO, USA. A commercially available stoichiometric hydroxyapatite (HAP-100) was a supplied by Taihei Chemical Industrial Co. Ltd., Osaka, Japan. Furthermore, Sc_2_O_3_ was purchased from Kanto Chemical Co. Inc., Tokyo, Japan. In addition, ZrO_2_ were supplied from the Catalysis Society of Japan (Tokyo, Japan) as JRC-ZRO-7. All chemicals were used as received without purification.

### 2.2. Catalyst Preparations

A calcium-phosphorus hydroxyapatite catalyst (Ca-HAP) was prepared using hydrothermal methods [5,8]. First, a solution containing Ca(NO_3_)_2_·4H_2_O (132–238 mmol) in 564 mL of distilled water was added to a solution containing P_2_O_5_ (66 mmol) in 490 mL of distilled water with NaOH (460 mmol). Each preparation of the Ca/P molar ratio in the mixed solution was between 1.0 and 1.8. The resultant suspension was treated under hydrothermal conditions at 110 °C for 16 h with agitation in a Teflon-lined autoclave. After hydrothermal treatments, the resultant precipitates were washed repeatedly using centrifugal separation with distilled water; then they were dried overnight at 100 °C. 

Using an impregnation method, SiO_2_-supported P_2_O_5_ catalyst (P_2_O_5_/SiO_2_) was prepared. The 0.9 g of silica-gel was added to 2 mL of 0.5 molL^−1^ P_2_O_5_ aqueous solution. Then the mixture was stirred on a water bath until dry. The obtained material was dried further at 60 °C overnight. Before catalytic tests and characterizations, the prepared HAP catalysts were typically pretreated at 500 °C under vacuum or inert gas.

### 2.3. Catalyst Characterizations

Crystalline phases of as-prepared and used catalysts were identified using powder X-ray diffraction (XRD, Ultima IV; Rigaku Co., Tokyo, Japan) with Cu K radiation (40 kV, 20 mA). The prepared samples were observed using transmission electron microscopy (TEM, H-7000; Hitachi Ltd., Tokyo, Japan). The elemental compositions of hydroxyapatites were ascertained using inductively coupled plasma (ICP, ICPE-9000; Shimadzu Co., Kyoto, Japan). Approximately 4 mg of sample was dissolved in 10 mL of 0.1 M HNO_3_ solution; then it was diluted to 100 mL using distilled water. The ICP measurement error for Ca/P molar ratio was ±0.01. The specific surface areas of as-prepared and used catalysts were measured using nitrogen physisorption with the Brunauer-Emmett-Teller (BET) method (Belsorp Max; BEL Japan Inc., Osaka, Japan). The particle sizes of the prepared Ca-HAP samples were determined by the manual approach and calculated by averaging the diameters of ~150 particles in the TEM images. In addition, the particle sizes were also roughly estimated based on BET surface areas by assuming the density of 3.1 g cm^−3^ and spherical shape particles and also roughly calculated by using Scherrer’s equation based on X-ray diffraction peak of (002). The temperature-programmed desorption (TPD) of CO_2_ were carried out using a glass U-tube reactor equipped with a quadrupole mass spectrometer (CANON ANELVA M-201QA-TDM, Kawasaki, Japan). Samples (0.1 g) were preheated in He at 500 °C and exposure to a 10% CO_2_/He (50 kPa) gas at room temperature until saturation coverages were reached. Weakly adsorbed CO_2_ was removed by flushing with He at room temperature for 30 min. The temperature was then increased at a linear rate of 5 °C/min from 20 to 800 °C. The TPD spectra were normalized at the specific surface area of samples. The TPD of NH_3_ was carried out by almost same method as that of TPD of CO_2_, except for using 10% NH_3_/He gas. The densities of acidic sites and basic sites were estimated respectively from the amounts of adsorbed NH_3_ and CO_2_, which had been determined by the isotherms at 250 °C using Belsorp Max instrument after the pretreatments of catalysts at 430 °C for 3 h under vacuum. The temperature of 430 °C was maximum for a heating oven attached with the instrument.

### 2.4. Catalytic Reaction

Catalytic conversions of 1,6-hexanediol were typically conducted at 375 °C using 0.1 g of catalysts in a fixed-bed continuous-flow glass reactor (8 mm i.d.) under atmospheric pressure. After the sample powders were pelletized and crushed to the desired size (250–500 μm) to avoid pressure gradients in reactor, they were pretreated at 500 °C for 3 h in N_2_ flow before catalytic reactions. Then 1,6-hexanediol was diluted by ethanol (1,6-hexanediol concentration was 10 mol%). The mixed solution was introduced into the reactor using a syringe pump with flowing N_2_ (30 mL min^−1^). The reaction products were condensed in a cold ethanol trap at −50 °C. The collected liquid samples and gas samples at the outlet of the trap were analyzed by using a GC-FID (GC-2014; Shimadzu Co., Kyoto, Japan) With a DB-wax capillary column (30 m, 0.32 mm) and a GC-MS (6890N/5973N; Agilent Technologies, Inc., Santa Clara, CA, USA) with a DB-1 capillary column (60 m, 0.25 mm). The 1,6-hexanediol conversions, product yields, and product selectivities were calculated based on the following equations.

$$1,6-\mathrm{Hexanediol}\;\mathrm{conversion}\;(C-\%)\;=\;(1\;-\;\frac{C\;\mathrm{mmol}\;\mathrm{unreacted}\;1,6-\mathrm{hexanediol}}{C\;\mathrm{mmol}\;\mathrm{of}\;\mathrm{introduced}\;1,6-\mathrm{hexanediol}})\;\times \;100$$

$$\mathrm{Product}\;\mathrm{yield}\;(C-\%)\;=\frac{C\;\mathrm{mmol}\;\mathrm{of}\;\mathrm{product}}{C\;\mathrm{mmol}\;\mathrm{of}\;\mathrm{introduced}\;1,6-\mathrm{hexanediol}}\;\times \;100$$

$$\mathrm{Product}\;\mathrm{selectivity}\;(C-\%)\;=\;\frac{\mathrm{Product}\;\mathrm{yield}}{1,6-\mathrm{hexanediol}\;\mathrm{conversion}}\;\times \;100$$

The mass balance was calculated as the ratio of the weight of collected liquid to the weight of introduced solution. The mass balance in ethanol conversion over the Ca-HAP catalysts was 100 ± 1%.

The catalytic conversions of 2-propanol were conducted using a fixed-bed continuous-flow glass reactor (10 mm i.d.) under atmospheric pressure to estimate the acid–base catalytic properties. Vapor of 2-propanol was introduced into the reactor with flowing N_2_. The total flow rate was 30 mL min^−1^. The partial pressure of 2-propanol was 960 Pa. The reaction products were analyzed using GC-FID (GC-2010; Shimadzu Co., Kyoto, Japan) with a Stabilwax capillary column (30 m, 0.32 mm).

$$\mathrm{Acetone}\;\mathrm{selectivity}\;(C-\%)\;=\;\frac{C\;\mathrm{mmol}\;\mathrm{of}\;\mathrm{acetone}}{C\;\mathrm{mmol}\;\mathrm{of}\;\mathrm{acetone}\;+\;C\;\mathrm{mmol}\;\mathrm{of}\;\mathrm{propylene}}\;\times \;100$$

## 3. Results

### 3.1. Characterization

Calcium-phosphorus hydroxyapatite catalyst (Ca-HAP) was prepared by the hydrothermal method at 110 °C using sodium hydroxide as an alkaline source. The initial Ca/P molar ratios in the mixed solution were between 1.0 and 1.8. The pH of the filtrates after the preparations was 12.2–12.8. Table 1 shows the chemical compositions and the specific surface areas of the prepared Ca-HAP catalysts. In the case of initial Ca/P ratios lower than 1.66, the resulted Ca/P ratios of particles were higher than the initial Ca/P ratios. This tendency was similar to the cases where ammonia was used as an alkali source without sodium species [15]. Whereas in the case of initial Ca/P ratios higher than 1.67, the resulted Ca/P ratios of particles were lower than the initial Ca/P ratios. The Ca/P molar ratios of particles were 1.54–1.72.

**Figure 1:**
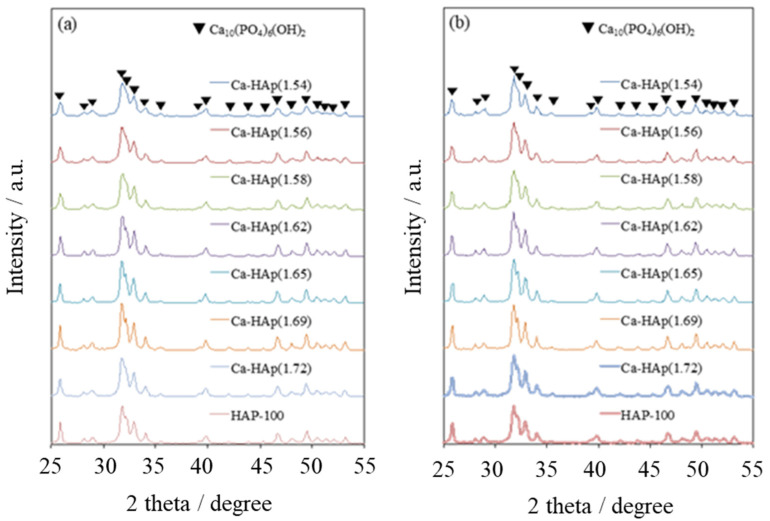
Powder X-ray diffraction (XRD) patterns of hydroxyapatites (**a**) as-prepared and (**b**) after conversion of 1,6-hexanediol.

As shown in Table 1, the specific surface areas of the prepared Ca-HAP catalysts were 51–79 m ^2^ g ^−1^. Figure 1a shows powder XRD patterns of prepared Ca-HAP catalysts. All XRD patterns were attributable to hydroxyapatite Ca_5_(PO_4_)_3_OH (JCPDS #01-074-0506) with a single phase. The peak intensities were almost identical, suggesting that the prepared catalysts had siTmilar crystallinity. In previous reports, Ca(OH)_2_, which coexists in Ca-HAP, with Ca/P of 1.7 or more were considered [1,27]. In this study, hydroxyapatite particles with a Ca/P ratio of 1.7 were obtained to be a single phase by the hydrothermal method. It was considered that Ca-HAP(1.72) could be a solid solution resulting from the partial replacement of PO_4_^3−^ ions with CO_3_^2−^ ions [1,27]. In consequence, according to the XRD patterns, it was observed that single-phase hydroxyapatite particles were synthesized with a Ca/P molar ratio of 1.54–1.72.

**Figure 2:**
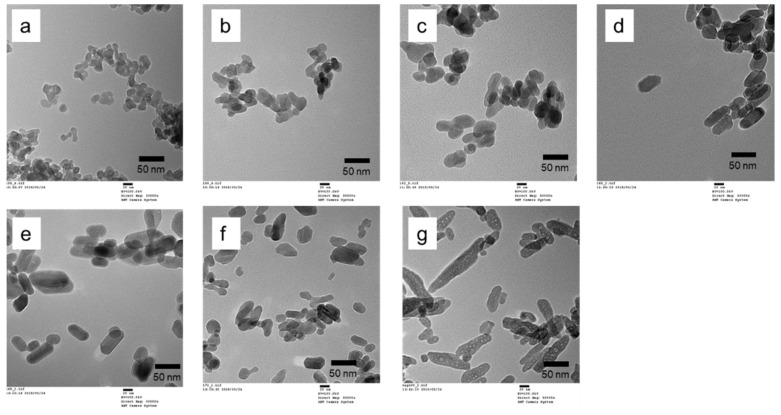
Transmission electron microscopy (TEM) images of the Ca-HAP catalysts. (**a**) Ca-HAP(1.54), (**b**) Ca-HAP(1.58), (**c**) Ca-HAP(1.62), (**d**) Ca-HAP(1.65), (**e**) Ca-HAP(1.69), (**f**) Ca-HAP(1.72), and (**g**) commercially available hydroxyapatite (HAP-100).

Some TEM images of the Ca-HAP catalysts are presented in Figure 2. All prepared Ca-HAP hydroxyapatites were column particles with average lengths of 24–45 nm. As shown in Table 2, the average widths were 14–27 nm. The particle sizes increased concomitantly with increasing Ca/P ratios, except for Ca-HAP(1.72). Table 2 also shows the particle sizes obtained by a simple calculation assuming the density of 3.1 g cm^−3^ and spherical shape particles. The particle sizes were 25–39 nm and were closed to the particle sizes observed by TEM. These results indicated that the particles observed by TEM were average values reflecting the whole of particles. Table 2 also shows particle sizes of Ca-HAP catalysts roughly calculated by using Scherrer’s equation based on XRD patterns presented in Figure 1 The particle sizes were 25–40 nm and were almost equal to those estimated with TEM, which implied that the particles observed in TEM images were single crystals. In consequence, the prepared Ca-HAP catalysts with various Ca/P molar ratios of 1.54–1.72 had almost identical crystallinity of hydroxyapatite structure to that of single-phase materials, with particle sizes of about 20–40 nm.

As shown in Table 1, Na^+^ ions were detected in the prepared Ca-HAP catalysts with Ca/P molar ratios less than 1.65. The Na^+^ contents increased concomitantly with decreasing Ca/P molar ratios. In addition, the molar ratios of (Ca+Na)/P in the prepared resulted Ca-HAP particles except for Ca-HAP(1.72) were 1.67–1.69, which were closed to stoichiometric ratio of 1.67. These results showed agreement with earlier reports, suggesting that Na^+^ ions might replace the Ca^2+^ deficiency in the Ca-HAP catalysts with low Ca/P ratios [13]. Because the ion radius of Na^+^ ion is close to that of Ca^2+^ ions and there were almost the same amounts of Na^+^ ions as those of the deficient Ca^2+^ ions in the Ca-HAP prepared by the hydrothermal method using NaOH, Na^+^ ions might occupy the calcium-deficient vacancy-sites in the Ca-HAP particles. Matsunaga and Murata reported that the substitution of Ca^2+^ ions in Ca-HAP by Na^+^ ions with charge-compensating interstitial H^+^ ions was energetically favorable, and that the H^+^ ion is attached to the OH group to form an H_2_O group [28]. Based on this first principle calculation investigation, the Ca-HAP particles could be expressed as Ca_10−n_Na_n_(PO_4_)_6_(OH)_2−n_(H_2_O)_n_.

### 3.2. Characterization

The adsorption amounts of NH_3_ and CO_2_ on the Ca-HAP catalysts at 250 °C were measured. Before the adsorption measurements, the catalyst was pretreated at 500 °C which was the same temperature as the pretreatment of catalytic reactions. The XRD measurement and BET specific surface area were almost the same as the post-reaction data shown in Figure 1b and Table 1, and no crystal phase other than hydroxyapatite was observed, the crystallite size increased slightly, and the specific surface area decreased by about 20%. As shown in Table 3, the NH_3_ adsorption amounts increased slightly with the increase of Ca/P molar ratios. The CO_2_ adsorption amounts increased greatly with the increase of Ca/P molar ratios. Figures S1 and S2, respectively, portray NH_3_-TPD and CO_2_-TPD of the Ca-HAP catalysts. The NH_3_-TPD intensity increased at around 250 °C with the increase of the Ca/P molar ratios. The CO_2_-TPD intensity at around 250 °C also increased concomitantly with the increase of the Ca/P molar ratios. The results corresponded with the adsorbed NH_3_ and CO_2_ amount data presented in Table 3, indicating that both the basic site density and acidic site density increased concomitantly with increasing Ca/P molar ratios of the Ca-HAP catalysts.

Table 4 shows the 2-propanol conversions over the prepared Ca-HAP catalysts, P_2_O_5_/SiO_2_, Ca(OH)_2_ catalysts. The specific surface area of each catalyst is presented in Table S1. Acetone and propylene were formed as products. Over Ca-HAP catalysts, CO and CO_2_ were not detected. The product selectivity was constant with time on stream for at least 20 h [5]. The selectivity of propylene was 100% over acidic catalysts as P_2_O_5_/SiO_2_. In contrast, the acetone selectivities of basic catalysts as Ca(OH)_2_ were, respectively, 90% and 99%. The acidic catalyst showed almost 100% of the propylene selectivity. The basic catalyst showed 90% or more of the acetone selectivity. However, the acid–base bifunctional Ca-HAP catalysts with low Ca/P molar ratio showed high selectivity of propylene. Moreover, the acetone selectivity increased concomitantly with increase of the Ca/P molar ratio. These results indicate that Ca-HAP catalysts with a low Ca/P molar ratio exhibited high acidity; the basicity became dominant instead of acidity as the Ca/P molar ratio increased. According to Figure 2 and Table 4, the morphology effects of Ca-HAP catalyst particles on the product selectivities were almost negligible. Table 3 shows that the specific ratios of the basic site density to the acidic site density increased concomitantly with increasing Ca/P molar ratios. The specific ratios of the basic site density to the acidic site density showed good agreement with the acetone selectivity in the 2-propanol conversion. The results suggest that the Ca-HAP catalysts with higher Ca/P molar ratios had higher basic properties to accelerate the dehydrogenation of hydroxy groups of alcohols into ketones and aldehydes preferentially over dehydration into unsaturated compounds.

### 3.3. Catalytic Conversion of 1,6-Hexanediol over Ca-HAP Catalysts

**Figure 3:**
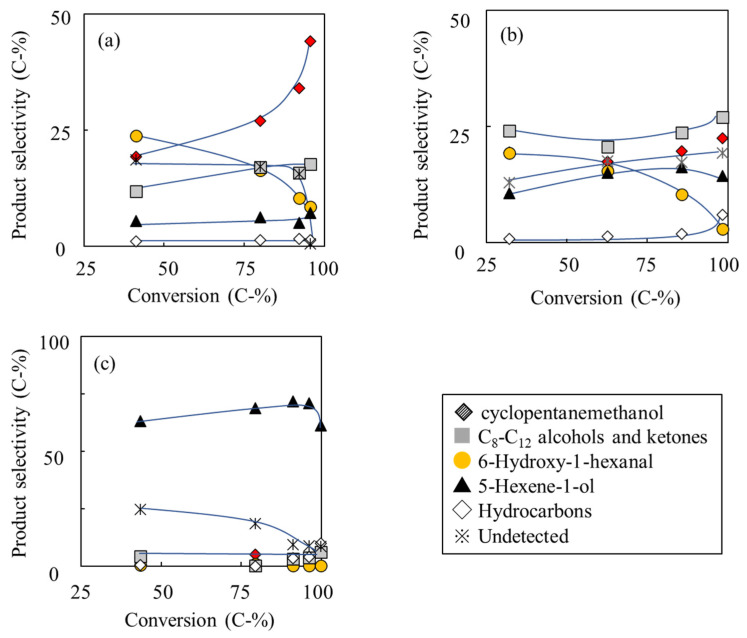
Relation between products selectivity and 1,6-hexanendiol conversion over Ca-HAP catalysts with various Ca/P molar ratios: (**a**) Ca-HAP(1.72), (**b**) Ca-HAP(1.62), and (**c**) Ca-HAP(1.54). Temperature 375 °C; 1,6-hexanediol 10 mol% EtOH solution, 1 mL h^−1^; N_2_, 30 mL min^−1^. Conversion and selectivity were averaged for the initial 3–5 h.

Catalytic conversions of 1,6-hexanediol over Ca-HAP catalysts with various Ca/P ratios were carried out using a fixed bed reactor at 375 °C (Scheme 1). Before reaction, the catalysts were pretreated at 500 °C under flowing nitrogen. Changes in major product selectivities against the 1,6-hexanediol conversions over Ca-HAP(1.72), Ca-HAP(1.62), and Ca-HAP(1.54) catalysts are presented in Figure 3. The major products were cyclopentanemethanol and 5-hexen-1-ol, and the by-products were 6-hydroxy-1-hexanal, C_8_-C_12_ alcohols and ketones, and hydrocarbons. In addition, acetaldehyde and 1-butanol were formed as by-products derived from ethanol. Ethanol was a solvent and a hydrogen source and was introduced nine times more than 1,6-hexanediol. However, the total yields of by-products from ethanol, such as acetaldehyde and 1-butanol, were about 2 C-% or less based on 1,6-hexanediol, even when those from 1,6 hexanediol were over 90%. These results implied that 1,6-hexanediol was much more reactive than ethanol on Ca-HAP catalysts.

As portrayed in Figure 3a for Ca-HAP(1.72) catalyst, the main product was cyclopentanemethanol. It increased concomitantly with increasing of conversion up to 96%. The C_8_-C_12_ oxygenated compound selectivity also increased. In contrast, the selectivities of 6-hydroxy-1-hexanal and the undetected compounds decreased concomitantly with increase of the conversion. The selectivities of 5-hexen-1-ol and hydrocarbons, respectively, recorded as 6% and 1%, were almost constant. As portrayed in Figure 3b for Ca-HAP(1.62) catalyst, the selectivities of the C_8_-C_12_ oxygenated compounds, cyclopentanemethanol, and hydrocarbons increased concomitantly with increase of the 1,6-hexanediol conversion. In contrast, the selectivities of 6-hydroxy-1-hexanal and 5-hexene-1-ol decreased concomitantly with increasing conversion. Figure 3c shows that the product selectivities of Ca-HAP(1.54) catalyst were less dependent on the conversion than Ca-HAP(1.72) and Ca-HAP(1.62) catalysts. The main product over Ca-HAP(1.54) catalyst was 5-hexene-1-ol. The 5-hexene-1-ol selectivity decreased concomitantly with increase at conversion of more than 90%. Instead, the hydrocarbon selectivity increased.

Over Ca-HAP(1.72) and Ca-HAP(1.62) catalysts, the selectivity of cyclopentanemethanol and the C_8_-C_12_ oxygenated compounds increased concomitantly with increasing conversion. Instead, the 6-hydroxy-1-hexanal selectivity decreased. Those results indicate that 6-hydroxy-1-hexanal is an intermediate of the formations of cyclopentanemethanol and the C_8_-C_12_ deoxygenated compounds. Cyclopentanemethanol formation was found to be predominant over Ca-HAP(1.72), whereas the formations of the C_8_-C_12_ deoxygenated compounds were predominant over Ca-HAP(1.62). The selectivity difference was regarded as attributable to the stronger basic properties of Ca-HAP(1.72) to accelerate the intramolecular aldol reaction. However, over Ca-HAP(1.54) catalyst, the dehydration reaction to 5-hexen-1-ol became dominant. In addition, Table S2 presents results of catalytic conversion of 5-hexen-1-ol over Ca-HAP(1.54) under almost the same reaction conditions. The 5-hexen-1-ol conversion was 20%. This result indicated that 5-hexen-1-ol conversion was less reactive than 1,6-hexanediol conversion over Ca-HAP(1.54) catalyst. For that reason, Ca-HAP(1.54) showed high selectivity of 5-hexen-1-ol.

Table 5 presents results of 1,6-hexanediol conversions over the Ca-HAP catalysts and the other acidic, basic, and acid–base bifunctional catalysts at 375 °C. The loading amounts of catalysts were adjusted so that the conversions were 93–98%, except for P_2_O_5_/SiO_2_ catalysts. The conversion and product selectivity were approximately the same during 5 h of time on stream over the entire tested catalysts. The catalysts remained almost white after reactions, except for P_2_O_5_/SiO_2_.

Among the prepared Ca-HAP catalysts in Table 5, the selectivity of 5-hexen-1-ol was maximized using Ca-HAP(1.54) catalyst. The selectivity decreased concomitantly with decrease of the Ca/P molar ratio. By contrast, the highest cyclopentanemethanol selectivity was obtained using Ca-HAP(1.72) catalyst. The selectivity increased concomitantly with increasing Ca/P molar ratio. Furthermore, the products formed were 6-hydroxy-1-hexanal, oxepane, and other CPMs, i.e., 2-cyclopentenylmethanol and cyclopentacarbaldehyde, and CPNs, i.e., cyclopentanone, cyclopentanol, 2-methylcyclopentanone, and 2-methylcyclopentanol, C_8_-C_12_ oxygenated compounds, such as cycloheptanemethanol, 2-ethyl-1-hexanol, 6-undecanone, and C_4_-C_6_ hydrocarbons. The selectivities of other CPMs and CPNs increased concomitantly with increase of Ca/P molar ratio. A similar tendency was apparent: 6-hydroxy-1-hexanal selectivity was the lowest at 0.3% over Ca-HAP(1.54) and the highest at 8.5% over Ca-HAP(1.72). Conversely, the hydrocarbon selectivity was the highest at 4.0% over Ca-HAP1.54 and the lowest over Ca-HAP1.72. The selectivities of the C_8_-C_12_ oxygenated compounds were higher over Ca-HAP catalysts with medium Ca/P molar ratios as Ca-HAP(1.62). The specific catalytic activities per the specific surface areas among Ca-HAP catalysts were as follows:

Ca-HAP(1.69) > Ca-HAP(1.65), Ca-HAP(1.62), Ca-HAP(1.58) > Ca-HAP(1.54) > Ca-HAP(1.72)

Ca-HAP(1.69) with almost the stoichiometric Ca/P ratio had the highest catalytic activity per specific surface area, and the catalytic activity decreased with decreasing the Ca/P ratio decreases, and the catalytic activity of Ca-HAP(1.72) with a higher Ca/P ratio than the stoichiometry decreased by about half. The cause of the different catalytic activity was complicated, but it is considered that it is mainly due to the difference in product selectivity due to the difference in surface acid–base nature of the Ca-HAP catalysts with various Ca/P ratios. In addition, the lower the aspect ratios of the morphology and the smaller the particles, the lower the activity per specific surface area tended to be, suggesting that the morphology of Ca-HAP catalysts might also have an effect on the catalytic activity.

Table 5 also shows the catalytic properties of phosphoric acid and calcium hydroxide, which were the constituents of Ca-HAP catalysts. The P_2_O_5_/SiO_2_ catalyst showed about 60% selectivity to 5-hexene-1-ol and 20% selectivity to oxepane. Ca(OH)_2_ catalysts with specific surface area of 16 m^2^ g^−1^ (see Table S1) had low catalytic activity and required 3.0 g to obtain 90% conversion of 1,6-hexanediol. When the experiment was carried out even using 3.0 g of catalyst, we checked no pressure drop. Ca(OH)_2_ catalysts gave cyclopentanemethanol and 5-hexene-1-ol as products, but their selectivities were as low as less than 3%. In previous representative studies, Sato et al. reported that 5-hexen-1-ol selectivities in 1,6-hexanediol conversion over Sc_2_O_5_ and ZrO_2_ were, respectively, 62% and 33% [24]. Sc_2_O_5_ and ZrO_2_ catalysts also showed acid–base catalytic properties as shown in Table 4. In our experiments for 1,6 hexanediol conversions over Sc_2_O_5_ and ZrO_2_ catalysts, 5-hexen-1-ol selectivities were, respectively, 61% and 37%, which showed good agreement with reported results. The results revealed that Ca-HAP(1.54) catalyst have the both acid–base bifunctionality similar to Sc_2_O_3_ and ZrO_2_ catalysts and the significant higher catalytic selectivity for unsaturated alcohol productions.

In consequence, over acidic catalysts, C_4_–C_6_ hydrocarbons and 5-hexene-1-ol were obtained as major products. Over base catalysts, many kinds of five-membered and six-membered cyclic compounds and 6-hydroxy-1-hexanal were obtained. Over Ca-P hydroxyapatite catalyst, 5-hexen-1-ol and cyclopentanemethanol were mainly obtained. Remarkably, Ca-HAP(1.72) catalyst showed the highest cyclopentanemethanol yield ever reported. Furthermore, the Ca-HAP(1.54) catalyst showed the highest 5-hexene-1-ol yield ever reported. The next section describes details of an investigation of the effects of Ca/P molar ratios on product selectivities over Ca-HAP catalysts.

### 3.4. Effects of Acid–Base Properties of Catalysts on the Products Selectivity

**Figure 4:**
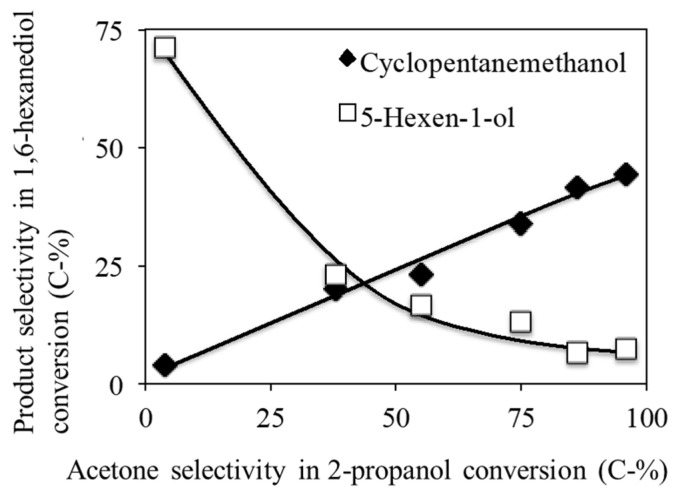
Effects of acid–base property on the products selectivity in the 1,6-hexanediol conversion over the Ca-HAP catalysts with various Ca/P molar ratios.

Good correlation was found between the acid–base properties and the product selectivity in the 1,6-hexanediol conversion over Ca-HAP catalysts with various Ca/P molar ratios. Figure 4 presents the relation between the acid–base properties of Ca-HAP catalysts and the product selectivity for 5-hexen-1-ol and cyclopentanemethanol. The acid–basic properties of Ca-HAP catalysts were estimated based on the selectivities of acetone and propylene in the 2-propanol conversions shown in Table 3. Among Ca-HAP catalysts, the cyclopentanemethanol selectivity increased along with the increase of the acetone selectivity. In contrast, the 5-hexen-1-ol selectivity increased concomitantly with the decrease of acetone selectivity because the higher acidity of Ca-HAP catalyst showed higher selectivity of dehydration to 5-hexen-1-ol from 1,6-hexanediol. The Ca-HAP catalysts had little catalytic activity for 5-hexen-1-ol conversion. A good correlation was found between the product selectivity of acetone and propylene in the 2-propanol conversion and the product selectivity of 5-hexen-1-ol and cyclopentanemethanol in the 1,6-hexanediol conversion. The Ca-HAP(1.54) showed high 5-hexene-1-ol yield from 1,6-hexanediol, whose catalytic properties were affected by the Ca/P ratios and the Na amounts but hardly affected by the morphology. The Ca-HAP(1.54) catalyst also showed high acrylic acid yields in the lactic acid conversion. It was probably suitable for the formation of unsaturated oxygen-containing compounds. Further details will be clarified in future research. In addition, Ca(OH)_2_ catalysts with high basicity gave high selectivity to by-products because of insufficient activity to proceed with the hydrogenation steps of the intermediates. The Ca-HAP catalysts with middle Ca/P molar ratios as Ca-HAP(1.62), which had moderate acid–base properties, also gave high selectivity to by-products because of the stronger selectivity to 5-hexen-1-ol and insufficient activity for the hydrogenation steps.

## 4. Conclusions

Nanocrystalline hydroxyapatite catalysts with various Ca/P ratios and sodium contents were formed by the hydrothermal method. According to adsorption and desorption experiments using NH_3_ and CO_2_ molecules and the catalytic reactions for 2-propenol conversions, the surface acid–base natures changed continuously with the bulk Ca/P ratios. In 1,6-hexanediol conversion over Ca-P hydroxyapatite catalysts, 5-hexen-1-ol, and cyclopentanemethanol were mainly obtained. Remarkably, those product selectivities were changed considerably with changing Ca/P molar ratios of Ca-HAP catalysts. The selectivity of 5-hexen-1-ol was maximized over Ca-HAP(1.54). The yield was 68%. However, the highest cyclopentanemethanol selectivity was obtained over Ca-HAP(1.72): the yield was 42%. Both yields were the highest ever reported in the relevant literature. The reason was presumably that Ca-HAP(1.72) mainly had basic properties and only slightly acidic properties.

## Figures and Tables

**Table 1:** Characterizations of prepared hydroxyapatite catalysts.

	Initial Solution	Resulted Particles			Surface Area/m^2^ g^−1^		pH
	Ca/P Molar Ratio	Ca/P Molar Ratio	Na Content wt%	(Ca+Na)/P Molar Ratio	As-Prepared	Used in 1,6-Hexanediol Conversion	After Preparation
Ca-HAP(1.54)	1	1.54	2.3	1.69	79	41	12.9
Ca-HAP(1.58)	1.5	1.58	1.7	1.69	65	46	12.8
Ca-HAP(1.62)	1.55	1.62	0.8	1.67	52	41	12.5
Ca-HAP(1.65)	1.67	1.65	0.4	1.67	52	40	12.2
Ca-HAP(1.69)	1.72	1.69	0.0	1.69	50	38	12.1
Ca-HAP(1.72)	1.8	1.72	0.0	1.72	65	55	11.0

**Table 2:** Average particle sizes of prepared Ca-HAP particles.

-	Average Particle Sizes (TEM)		Average Particle Sizes (BET)	Average Particle Sizes (XRD)
	Length/nm	Width/nm	nm	nm
Ca-HAP(1.54)	24	14	25	25
Ca-HAP(1.58)	29	19	28	30
Ca-HAP(1.62)	37	24	36	37
Ca-HAP(1.65)	39	25	33	37
Ca-HAP(1.69)	45	27	40	39
Ca-HAP(1.72)	34	20	29	30

**Table 3:** Adsorption amounts of NH_3_ and CO_2_ on Ca-HAP catalysts with various Ca/P molar ratios.

Sample	Adsorption Amount of NH_3_ (μmol m^−2^)	Adsorption Amount of CO_2_ (μmol m^−2^)
Ca-HAP(1.72)	3.93	1.62
Ca-HAP(1.65)	3.45	1.58
Ca-HAP(1.58)	3.12	0.95
Ca-HAP(1.54)	2.65	0.25

**Table 4:** Catalytic conversion of 2-propanol into propylene and acetone.

Catalyst	Selectivity (C-%)	
	Propylene	Acetone
Ca-HAP(1.54)	96	4
Ca-HAP(1.58)	62	38
Ca-HAP(1.62)	45	55
Ca-HAP(1.65)	23	77
Ca-HAP(1.69)	14	86
Ca-HAP(1.72)	4	96
HAP-100	25	75
P_2_O_5_/SiO_2_	100	0
Ca(OH)_2_	1	99
Sc_2_O_5_	40	60
ZrO_2_	95	5

With a 250 °C reaction temperature, 980 kPa; 2-propanol partial pressure, 30 mL min^−1^; N_2_ flow, 0.05–0.2 g; catalyst, 0.3–5.8%; Conversion (100% conversion over P_2_O_5_/SiO_2_).

**Table 5:** 1,6-hexanediol conversion over various catalysts.

Catalyst	Catalyst Weight (g)	Conversion (C-%)	Selectivity (C-%)								
			Hydro Carbons	Oxepane	5-Hexen -1-ol	Cyclopentanemethanol	Other CPMs ^a)^	CPNs ^b)^	6-Hydroxy -1-hexanal	C_8_-C_12_ -OH, CO ^c)^	Others (Undetected)
Ca-HAP(1.54)	0.26	96.2	4.0	8.0	71.0	3.9	0.1	0.3	0.3	3.7	8.6
Ca-HAP(1.56)	0.20	93.5	3.9	4.9	38.7	16.2	3.5	1.0	5.6	23.5	6.6
Ca-HAP(1.58)	0.20	93.1	1.7	2.3	23.0	19.9	2.1	0.7	3.3	24.5	22.6
Ca-HAP(1.62)	0.20	98.3	5.6	3.4	14.9	22.4	3.1	1.9	2.9	27.0	18.9
Ca-HAP(1.65)	0.20	93.9	2.8	3.5	13.8	34.7	5.0	1.4	5.7	17.9	15.2
Ca-HAP(1.69)	0.15	97.5	3.8	2.6	6.5	41.7	3.6	2.7	3.1	19.7	16.4
Ca-HAP(1.72)	0.40	95.6	1.3	4.8	7.2	44.2	9.7	6.1	8.5	17.7	0.6
HAP-100	0.18	98.0	2.6	3.2	22.3	26.1	1.7	1.9	0.9	32.2	9.1
P_2_O_5_/SiO_2_	0.20	44.5	1.0	18.8	59.6	0.0	0.0	0.0	0.0	0.0	20.6
Ca(OH)_2_	3.00	89.7	1.3	1.1	3.0	15.7	2.5	15.4	11.7	16.4	32.8
Sc_2_O_3_	0.30	93.3	0.7	0.3	61.4	0.4	0.0	0.5	0.0	0.0	36.7
ZrO_2_	0.20	97.8	11.9	14.8	36.7	0.4	0.0	0.0	0.0	0.0	36.2

375 °C temperature, 1.0 mL h^−1^; 1,6-hexanediol 10 mol%; EtOH solution, 30 mL min^−1^; N_2_, 5 h time on stream. ^a)^ Other CPMs; 2-cyclopenthenylmethanol+cyclopentacarbaldehyde. ^b)^ CPNs; cyclopentanone+cyclopentanol+2-methyl cyclopentanone+2-methyl cyclopentanonol. ^c)^ C_8_-C_12_-OH, -CO; C_8_-C_12_ oxygenated compounds such as cycloheptanemethanol, 2-ethyl-1-hexanol, and 6-undecanone.

## Supplementary Materials

The following are available online at https://www.mdpi.com/2079-4991/11/3/659/s1. Table S1: Surface areas of solid catalysts; Table S2: Catalytic conversion of 5-hexen-1-ol over Ca-HAP(1.54); Figure S1: NH_3_-TPD spectra of hydroxyapatites; Figure S2: CO_2_-TPD spectra of hydroxyapatites.
