# Supplementary material for: The Role of the Surface Acid–Base Nature of Nanocrystalline Hydroxyapatite Catalysts in the 1,6-Hexanediol Conversion
Source: Nanomaterials (Basel). 2021 Mar 8;11(3):659. doi: 10.3390/nano11030659 (PMC8000547; doi:10.3390/nano11030659)
Supplement: Supplementary file 1 [file nanomaterials-11-00659-s001.pdf]

Supplementary Materials

The Role of the Surface Acid–Base Nature of Nanocrystalline Hydroxyapatite Catalysts in the 1,6-Hexanediol Conversion

Asato Nakagiri, Kazuya Imamura, Kazumichi Yanagisawa and Ayumu Onda *

Research Laboratory of Hydrothermal Chemistry, Faculty of Science, Kochi University, 2-17-47 Asakurahonmachi, Kochi, 780-8073, Japan; nkgrpon3@gmail.com (A.N.); imamura-kazuya@kochi-u.ac.jp (K.I.); yanagi@kochi-u.ac.jp (K. Y.)

* Correspondence: aonda@kochi-u.ac.jp; Tel.: +81-88-8448353

List of Table Titles and Figure Captions of Supplementary materials

Table S1. Surface areas of solid catalysts

Table S2. Catalytic conversion of 5-hexen-1-ol over Ca-HAP(1.54)

Figure S1. NH_3-TPD spectra of hydroxyapatites

Figure S2. CO_2-TPD spectra of hydroxyapatites

Table S1. Surface areas of solid catalysts

Catalyst	Specific surface area ($m^2 g^{-1}$)	
	before reaction	after reaction
P_2O_5 / SiO_2	143	63.0
$Ca(OH)_2$	-	15.8
Sc_2O_5	15	14.1
ZrO_2	-	7.4

Table S2. Catalytic conversion of 5-hexen-1-ol over Ca-HAP(1.54)

Time on stream	conversion(%)	Selectivity(C-%)					
(h)	(C-%)	Hydrocarbons	5-Hexene ethyl ether	Cyclopentanemethan ol	6-Hydroxy-1-hexanol	1,6-hexanediol	Others(Unditected)
1	34.8	34.6	3.2	0.0	0.0	0.0	62.2
2	17.3	56.8	3.1	0.0	0.0	0.0	40.1
3	13.5	72.5	3.1	0.0	0.0	0.0	24.3
4	16.2	59.1	2.8	0.0	0.0	0.0	38.1
5	14.7	53.1	3.6	0.0	0.0	0.0	43.3

0.2 g catalyst weight, 375 °C reaction temperature, 500 °C · 3 h pretreatment temperature, 1.0 mL h^{-1} 5-hexen-1-ol 5 mol% EtOH solution, 30 mL min^{-1} N_2 flow rate

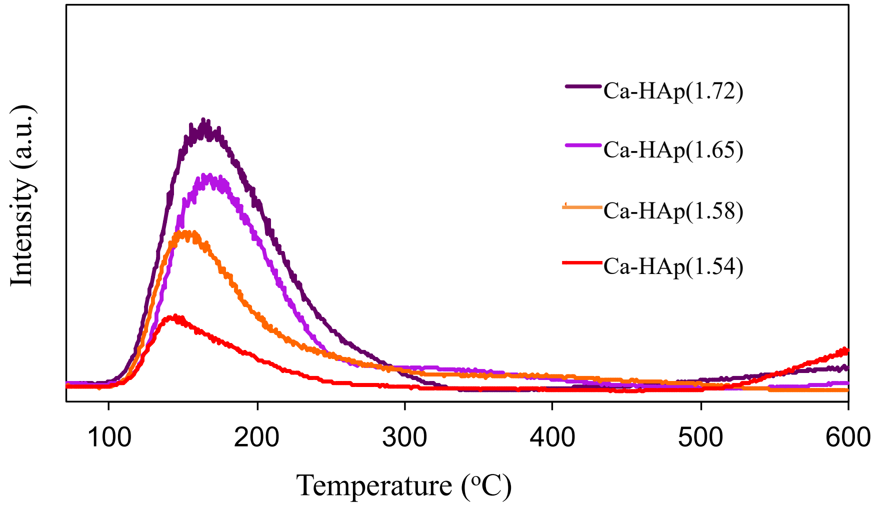

Figure S1 NH_3-TPD spectra of hydroxyapatites.

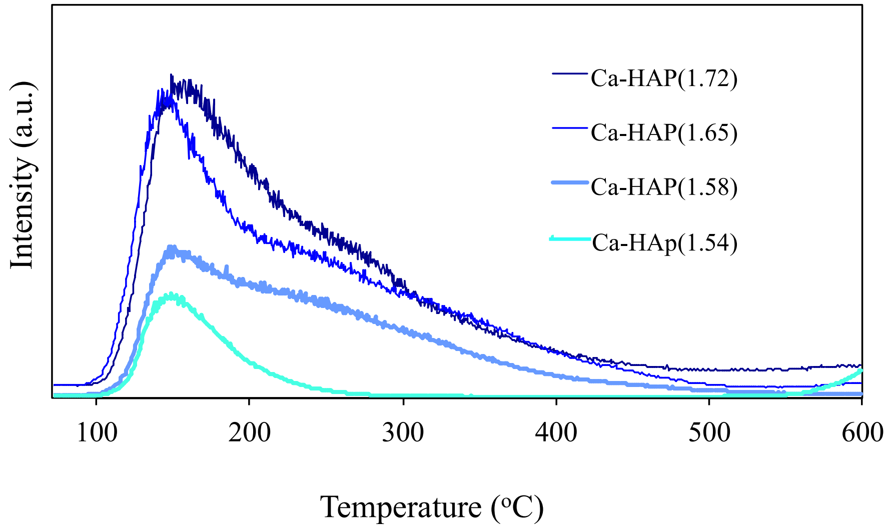

Figure S2 CO_2-TPD spectra of hydroxyapatites.
